# Hybrid Classical–Quantum Transfer Learning for Cardiomegaly Detection in Chest X-rays

**DOI:** 10.3390/jimaging9070128

**Published:** 2023-06-25

**Authors:** Pierre Decoodt, Tan Jun Liang, Soham Bopardikar, Hemavathi Santhanam, Alfaxad Eyembe, Begonya Garcia-Zapirain, Daniel Sierra-Sosa

**Affiliations:** 1 Cardiologie, Centre Hospitalo-Universitaire Brugmann, Faculté de Médecine, Université Libre de Bruxelles, Place Van Gehuchten 4, 1020 Brussels, Belgium; decpie@gmail.com; 2 School of Computer Science, Digital Health and Innovations Impact Lab, Taylor’s University, Subang Jaya 47500, Selangor, Malaysia; laolianglaoliang@gmail.com; 3 qBraid Co., Chicago, IL 60615, USA; 4 Department of Electronics and Telecommunication Engineering, College of Engineering Pune, Pune 411005, India; sohampb20.extc@coep.ac.in; 5 Faculty of Graduate Studies and Research, Saint Mary’s University, 923 Robie Street, Halifax, NS B3H 3C3, Canada; krishhema24@gmail.com; 6 Faculty of Engineering, Kyoto University of Advanced Science (KUAS), Ukyo-ku, Kyoto 615-8577, Japan; 2022m695@kuas.ac.jp; 7 eVIDA Research Group, Department of Engineering, Deusto University, 48007 Bilbao, Spain; mbgarciazapi@deusto.es; 8 Computer Science and Information Technologies Department, Hood College, 401 Rosemont Ave., Frederick, MD 21702, USA

**Keywords:** medical imaging, chest X-ray, diagnosis, cardiovascular diseases, heart failure, cardiomegaly, quantum computing, machine learning, transfer learning, visualization

## Abstract

Cardiovascular diseases are among the major health problems that are likely to benefit from promising developments in quantum machine learning for medical imaging. The chest X-ray (CXR), a widely used modality, can reveal cardiomegaly, even when performed primarily for a non-cardiological indication. Based on pre-trained DenseNet-121, we designed hybrid classical–quantum (CQ) transfer learning models to detect cardiomegaly in CXRs. Using Qiskit and PennyLane, we integrated a parameterized quantum circuit into a classic network implemented in PyTorch. We mined the CheXpert public repository to create a balanced dataset with 2436 posteroanterior CXRs from different patients distributed between cardiomegaly and the control. Using k-fold cross-validation, the CQ models were trained using a state vector simulator. The normalized global effective dimension allowed us to compare the trainability in the CQ models run on Qiskit. For prediction, ROC AUC scores up to 0.93 and accuracies up to 0.87 were achieved for several CQ models, rivaling the classical–classical (CC) model used as a reference. A trustworthy Grad-CAM++ heatmap with a hot zone covering the heart was visualized more often with the QC option than that with the CC option (94% vs. 61%, *p* < 0.001), which may boost the rate of acceptance by health professionals.

## 1. Introduction

According to the WHO’s Global Health Estimate [1], ischemic heart disease is the world’s biggest killer with 8.9 million victims in 2019, accounting for 16% of the world’s total deaths. Each year, about 700,000 persons in the United States and 1,700,000 persons in the 57 ESC member countries die from heart disease, i.e., about one in every five deaths [2,3]. Heart disease cost the United States about 229 billion USD each year from 2017 to 2018 and cost the European Union 210 billion EUR in 2015.

For this reason, cardiology has been one of the branches of medicine where the most progress has been made in recent years. The high prevalence of cardiovascular diseases has led to the constant development of new technologies for diagnosis and treatment. In recent decades, there has been growth in advanced technological developments applied to solving this situation addressing cardiovascular disease assessment, such as the application of Machine Learning (ML) algorithms for the diagnosis of heart failure [4] or cerebrovascular diseases [5], or for the analysis of arrhythmias [6,7]

Several deep learning techniques have been presented to assess cardiac diseases using both tabular data and images. This manuscript presents an approach that merges contributions from deep learning techniques with quantum ML techniques for detecting cardiomegaly in chest X-rays (CXR).

CXR is a radiological modality widely used in inpatient and outpatient settings. It is part of the tools used for assessment and follow-up in suspected or known cardiac patients. On the other hand, CXRs are more often performed for a purpose other than heart disease, e.g., in environments where they are first protocolized by on-duty physicians. Some CXR findings may indicate an unsuspected underlying heart condition, unrelated to the patient’s actual problem. Besides edema, pleural effusions and pulmonary opacity often revealing heart failure, cardiomegaly is the most relevant indicator of a cardiac problem. Pericardial effusion is another concerning cause of the characteristic enlargement of the heart silhouette, but its presence never excludes concomitant cardiomegaly. The two conditions can thus be aggregated in an early triage. Cardiomegaly is usually a sign of dilated or hypertrophic cardiomyopathy, which is observed in a large range of congenital and acquired diseases [8,9]. One prominent cause of cardiomyopathy is coronary artery disease [10].

Cardiomyopathy is a severe condition leading to heart failure, but also to sudden cardiac death, malignant arrhythmias and thromboembolism from cardiac origin. This represents a major cause of morbidity and mortality all over the world [11]. In summary, the initial radiological discovery of cardiomegaly may indicate, depending on the clinical context, the worsening of a known cardiac condition, an unsuspected cardiac disease or a cardiac complication during another disease. In all cases, a more in-depth cardiological assessment is indicated to clarify the diagnosis and establish appropriate therapeutic measures.

In this context, ML algorithms are being developed for the detection of cardiomegaly on radiographs. These studies are facilitated by the availability of large CXR databases, such as ChestX-ray8, CheXpert or MIMIC-CXR-JPG [12,13,14]. Encouraging results are reported. For instance, when comparing sixteen different deep learning architectures of CNN, ROC AUC scores up to 0.84 were found for the detection of cardiomegaly on a CXR validation dataset of 202 images from CheXpert [15].

Automated analyses of written protocols mention cardiomegaly in 9 to 17% of cases in these large CXR data sets. However, patients treated for heart disease are more likely to present cardiomegaly and undergo repetitive CXRs. For instance, in the CheXpert dataset, cardiomegaly is reported in 11% of the first studies against 13% of the follow-up studies. For medical applications, the performance indicators of a classification method must be such that there is little chance of missing a case while avoiding costly and resource-consuming additional diagnostic procedures. These considerations imply that a balanced dataset is a reasonable option for training the ML algorithm in such an application. Undersampling the majority class is indeed a good strategy that keeps the computational cost within reasonable limits [16], which was necessary to carry out the present study.

In less than a decade, deep learning has revolutionized the field of ML imaging, and automated CXR analyses can now benefit from these advances. Convolutional neural networks (CNN) have been the go-to pattern classification algorithms due to their well-demonstrated approximation and generalization capabilities. However, training a deep CNN in big data applications with a rich feature set is resource-intensive.

CNNs are based on the multiple perceptron model, in which a perceptron or an artificial neuron mimics the neurons in the human brain. In the human brain, specific regions of the brain are dedicated to responding to specific signals, e.g., images with faces trigger a stronger response than images without faces in a region called the fusiform face area (FFA), which is present on the underside of the temporal lobe. Further, in the middle layer of the brain’s visual cortex, specific neurons respond to specific shapes, edges, angles and layers [17]. This idea of specific neurons responding to specific features is used in the CNN model, in which each convolutional layer learns a specific feature/characteristic of the image using the image kernels and filters.

A CNN model can be viewed as two parts. The first part acts as a feature transformer that contains one or more layers that learn to identify and assign weights to the features of the image, and the second part acts as a classifier layer that classifies the images into different classes. Transfer learning is a useful technique that takes advantage of this two-part design, in which a model that was trained on a set of images is reused for training and classifying a completely different set [18]. The idea is to retain the convolutional layers that learn the common features of any image, such as lines, edges and curves, and to replace the classifier layers that are image-specific, thus reducing the resources required to fully train the model from the beginning.

This technique makes it possible to download CNNs pre-trained on thousands of image categories with millions of parameters. Using these models with their pre-trained weights, one can classify specific sets of images by retraining only the last parts of the model, where layers can be modified. The models pre-trained on colored images work for grayscale medical images, such as those of CT, mammography, MRI or CXR [19,20], and they have been developed for several conditions, including Alzheimer’s disease, brain tumors, breast tumors and COVID-19. For the automated detection of a set of abnormal findings in CXRs, transfer learning based on DenseNet-121 allows discrimination with satisfactory metric scores for most features [12,13]. Using 952 images from the National Institute of Health relabeled as “normal” or “heart failure” (defined as “cardiomegaly or congestion”) by two cardiologists, Matsumoto et al. [21] reported an accuracy of 82% in a model based on pre-trained VGG16.

Quantum neural networks were shown theoretically and experimentally to offer an advantage over their classical counterpart in terms of trainability [22], which justifies their use in medical applications [23]. To engineer a hybrid neural network, one can encode the images in an initial quantum layer, as in a classical–quantum model aimed at COVID-19 prediction presented in [24]. Another option is to insert the quantum layer instead of the classical one in the last part of a transfer learning model [25]. This CQ approach, proposed for detecting Alzheimer’s disease from brain CT scans [26] and stenosis detection in X-ray coronary angiography [27], was chosen to conduct this research.

A common problem with any diagnostic method based on deep learning is the opacity of the decision-making process, which hinders healthcare professionals’ and patients’ trust and acceptance [28,29]. Improved visual explanation by saliency methods, such as Grad-CAM++ [30], allows for locating the region of interest of the automated search process on a heatmap. For medical imaging, it is possible to check whether anatomically credible heatmaps can be produced by the algorithm. Cardiomegaly detection is particularly well-suited for this approach.

To the best of our knowledge, no description of a hybrid transfer learning CQ model for the detection of cardiomegaly in CXRs has been published so far. Our aim was to verify whether efficient CQ models can be designed using currently available software development kits (SDKs) and whether, compared to the CC approach, they can have advantages in terms of trainability, performance and trustworthiness. The objectives for carrying out this project include mining and balancing an adequate CXR dataset, selecting a performing reference model, inserting a parameterized quantum circuit (PQC) [31] in CQ models in place of the classical classifier layer and establishing a common training protocol. Two approaches were considered to assess the predictive performance. A 70/30 train–test split was used for model selection from numerous prototypes, for the comparison of training loss curves, and for the credibility assessment for saliency zones on GradCAM++ heatmaps. K-fold cross-validation, which is more computationally demanding, was used for possible overfitting detection, reference selection and QC vs. CC c statistical comparison of performance metrics. We also investigated in two Qiskit-based models the normalized global effective dimension (NGED), a parameter related to trainability and performance [22,32].

## 2. Materials and Methods

### 2.1. Dataset and Data Curation

The dataset was mined from the Stanford CheXpert shared dataset [33] consisting of 224,316 chest radiographs of 65,240 patients, reflecting the general CXR flow of a large Health Care Facility with both inpatient and outpatient centers. We aimed to establish an algorithm able to detect cardiomegaly in such a flow. The CheXpert dataset was selected because of the improved performance of the automated label extraction from free-text reports, especially for cardiomegaly [13]. The posteroanterior (PA) CXR view is recommended and has specific diagnostic criteria for cardiomegaly [34]. Therefore, we selected the images corresponding to the first chronological PA view in the source dataset. In the resulting reduced set of CXRs all from different patients, a subset of 1218 was labeled positive for cardiomegaly. A balanced dataset was formed by drawing, in the same reduced set, 1218 additional patients labeled negative (control subset).

One of us (Pierre Decoodt), a cardiologist with forty years of experience in the interpretation of CXR for heart disease, blindly reviewed the 2436 randomly presented images and requalified 111 of them, which were mislabeled “cardiomegaly” for 59 and “no cardiomegaly” for 52. In addition to clinical judgment, a cardiothoracic index lower or equal to 0.45 was required to requalify cardiomegaly as the control and greater or equal to 0.55 to requalify the control as cardiomegaly. For cases judged as doubtful, the labels remained intact. The rate of correct labels before requalification that we found was consistent with the labeler performance reported for the global CheXpert dataset, which was 0.973 for cardiomegaly and 0.909 for no cardiomegaly [13].

**Figure 1:**
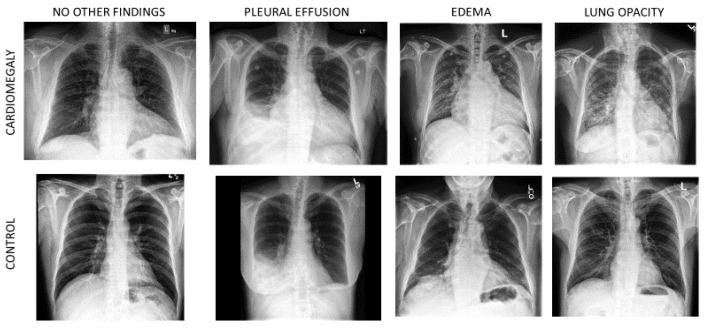
Images from the cardiomegaly subset along with their counterpart from the control subset. First column: no positive label for any other finding. Three last columns: cases of pleural effusion, edema and lung opacity, which were the findings most frequently associated with cardiomegaly in the dataset.

Table 1 describes the final dataset. All CXRs were from the general patient population studied at the Stanford Health Care Facility. This ensured that the control group was representative of the disease group, with no bias that could be introduced if the two classes were sampled from different populations. It can be observed that patients with positive labels for cardiomegaly were older. Male patients were the majority, but this was more marked in the control subset. In the whole dataset, 61% of patients presented one or several other abnormal CXR findings. Overall, the predominant conditions in the cardiomegaly subset included lung opacity, edema, pneumonia, atelectasis, pleural effusion or other pleural findings, and the presence of support devices, whereas pneumothorax was more frequent in control cases. Typical examples of CXR are illustrated in Figure 1.

### 2.2. General Design of the Models

**Figure 2:**
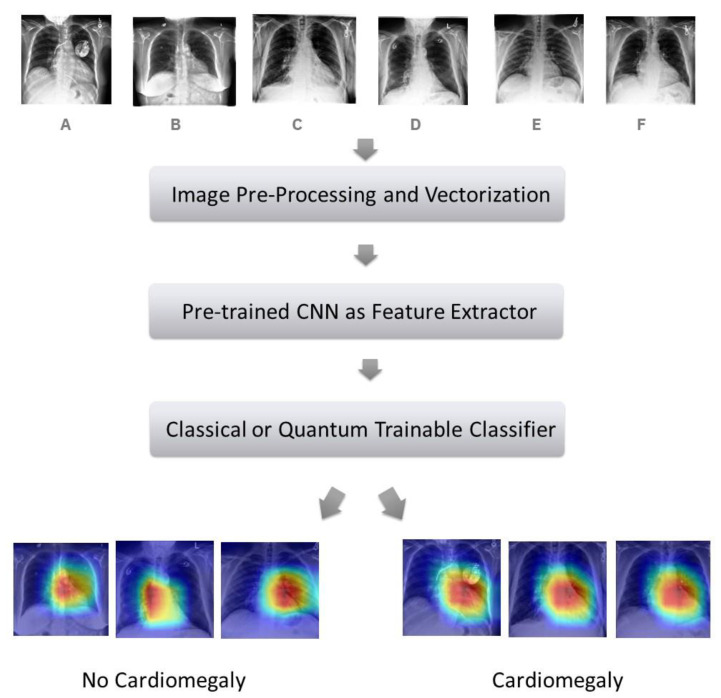
High-level model design. Six CXRs are represented (**A**–**F**) to describe the process output. Cardiomegaly is detected in (**A**,**C**,**F**).

Both models share the same architecture. The preprocessed and vectorized images were injected in a pre-trained CNN, used for extracting informative features. They then followed a trainable binary classifier and finally a sigmoid layer (Figure 2). The CQ version differed only by the presence of a PQC between two layers of the trainable classifier.

### 2.3. Image Preprocessing and Vectorization

The CXR images were resized to 256 × 256 pixels and then center-cropped to 224 × 224 pixels. They were then converted to tensor and normalized using the mean and standard deviation. The single-channel X-ray images originally in grayscale were transformed into a three-channel format by repeating the values in a particular image across all channels. This ensured compatibility with the tested pre-trained CNN.

### 2.4. Feature Extractor

A large selection of CNNs pre-trained on ImageNet 1 K V1 can be downloaded from PyTorch. After some preliminary tests with 8 training epochs, we selected AlexNet [35] and DenseNet-121 [36]. These two CNNs, renowned for their performance, differ greatly in their architecture, number of layers (8 versus 121), number of parameters (61.1 million vs. 8 million) and delivered feature vector *f* (4096 vs. 1024).

### 2.5. Trainable Classifier

**Figure 3:**
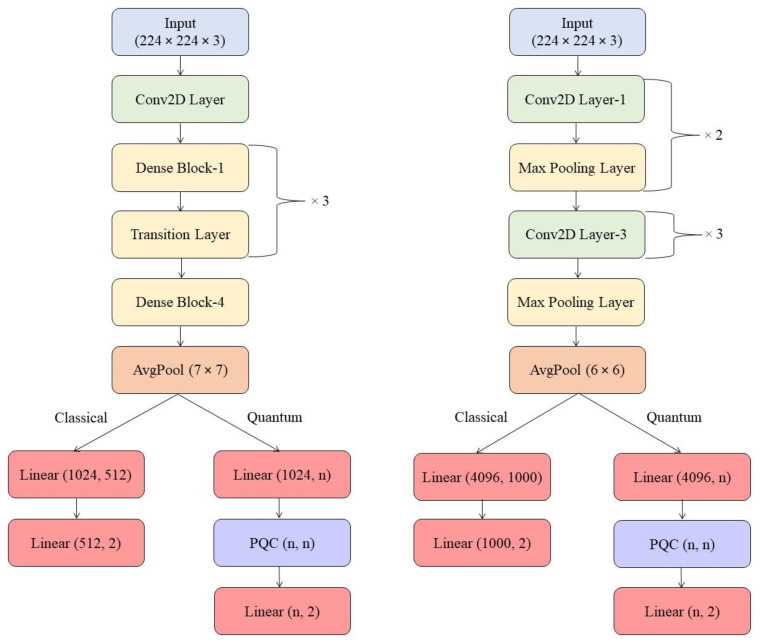
Training models for classification: On the left, a model based on pre-trained DenseNet-121. On the right, a model based on pre-trained AlexNet. In both versions, the flowchart forks into the classical and quantum versions of the trainable classifier. n: number of qubits.

In Figure 3 the classifiers of the CC and CQ models are depicted.

The trainable classical classifier consists of a sequence of linear (*f*, 512), ReLU and linear (512, 2) layers.

The trainable quantum classifier consists of a sequence of linear (*f*, n), tanh, *n*-qubit PQC and linear (*n*, 2) layers. For the Qiskit-based experiments, we tested 2 and 4 as the dimension output *D* for the classifier. Similarly, we tested 4, 6, 8 and 10 as *n* and *D* values for PennyLane-based models.

### 2.6. Parameterized Quantum Circuit

**Figure 4:**
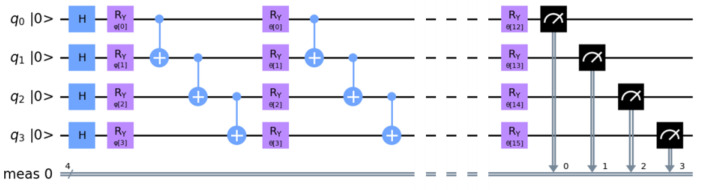
Qiskit rendering of the PQC with four qubits. After initialization in the ground state, all qubits are first placed in a superposition state by applying Hadamard gates (H). A feature map is produced by encoding each qubit by a φ rotation around the y-axis (Ry gates). Then, the ansatz consists of a series of entanglement by 2-qubit CNOT gates, each followed by a θ rotation around the *y*-axis at a quantum depth of 4.

To implement the hybrid classical–quantum models, we used the built-in methods of Qiskit and PennyLane, allowing us to integrate the PQC into the Pytorch framework. In Qiskit, we employed the CircuitQNN and TorchConnector functions from the Qiskit Machine Learning package. A four-qubit PQC is presented in Figure 4. The ansatz consists of repeated blocks of 2-qubit entanglement gates with a linear entanglement strategy and 1-qubit rotation gates (rotation angles being set by the circuit parameters).

### 2.7. Training

A 70/30 train–test split was applied to the dataset. We applied RandAugment and 50% RandomAutocontrast to the training data for each epoch.

All models were trained using the same random seed, with the learning rate and weight decay set to 0.0001, Cross-Entropy loss as the cost function and Adam [37] as the optimizer. We compared a standard and an enhanced protocol for training. The first consisted of 20 epochs without “freezer”. In the latter, this phase was preceded by training for 2 epochs with “freezer”, which froze the layer with pre-trained parameters acting like a weight initializer so that the further 18-epoch training was improved. The batch size was set to 8.

In Qiskit-based models, the computation was conducted in the Aer simulator statevector device with ten shots, using the default gradient settings [38]. In PennyLane-based models, we used as parameters the default qubit device, the torch interface and the “best” differentiation method. The latter depends on the device and interface, being in the most likely case Standard Backpropagation with Pauli-Z expectation values for each qubit [25].

### 2.8. Performance Metrics

The metrics we considered are listed in Table 2. All metric values mentioned in the following tables were for the test subsets. They were calculated using the corresponding functions from the metrics module of the open-access scikit-learn package [39]. The tables of results obtained from 10-fold cross-validation show the mean over the folds as well as, in brackets, the 95% confidence interval for the mean. For the comparison of a metric in two models, we performed a paired *t*-test on the values observed for each fold’s test dataset.

### 2.9. Image Interpretation

The image interpretation was visualized with a gradient-weighted class activation map using Grad-CAM++. To provide an idea of the acceptance by clinicians of the CC and CQ models, we grouped all the heatmaps obtained in the test set for each model and submitted them shuffled to one of us (Pierre Decoodt, a cardiologist), who blindly classified them as trustworthy or non-trustworthy. A heatmap was considered trustworthy if the hot zone comprised most of the cardiac silhouette. Heatmaps were classified as non-trustworthy when the hot zone was either outside the cardiac area, extremely extensive, or not visible. Heatmaps with multiple hot zones of similar size were also classified as non -trustworthy.

### 2.10. Normalized Global Effective Dimension

The trainability was assessed for the quantum layer on Qiskit by using the four-step algorithm that the qiskit-machine-learning package provides for assessing the NGED [40].

## 3. Results

### 3.1. Selection of Models and Training Protocols

Given the wide spectrum of possible combinations, we adopted a step-by-step heuristic to test model prototypes. We started by calculating the metrics using the 70/30 train–test split approach in thirteen models (Table 3).

As detailed in Appendix A, the results obtained by the single split approach (Table A1) were used for a preliminary analysis of the train and test ROC and loss curves. The calculation times are given in Table A2. The ROC curves for the train set in CC models led to suspecting a possible effect of overfitting that could affect the metric values observed in the test set. However, additional experimentation based on 10-fold cross-validation (Table A3) did not confirm this issue. The single-split results were used to select ten models for statistical comparison, to generate GradCAM++ heatmaps from the test subset and to perform NGED analysis.

The selected CC and CQ models were subjected to ten-fold cross-validation, which allowed us to obtain estimates of the mean and its 95% confidence interval for the metrics. This more computationally demanding technique was used to choose the CC model used as a reference, to investigate a possible overfitting effect (Table A3) and to establish the performance of CQ models that might challenge the reference.

### 3.2. Performances of the CC Models

**Figure 5:**
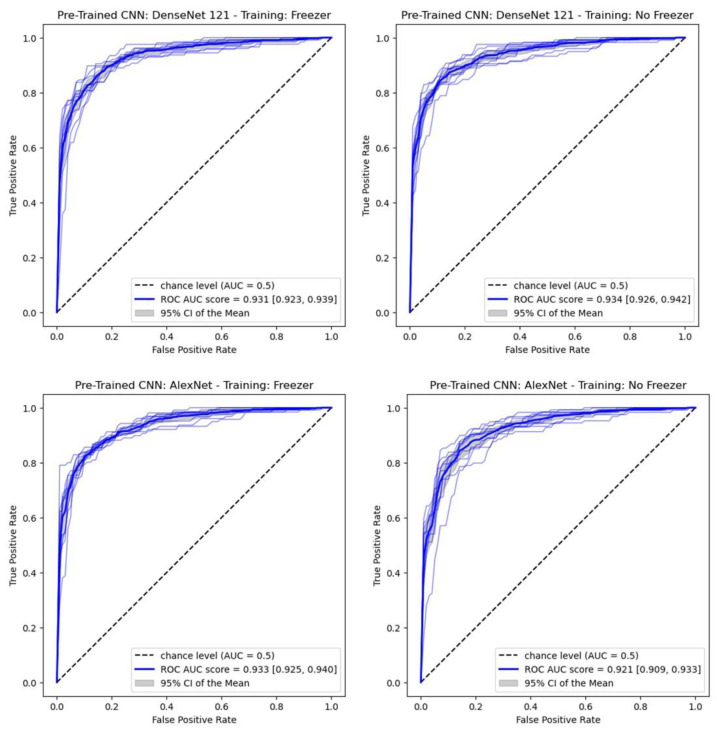
ROC curves obtained by 10-fold cross-validation in four CC models (test set).

Table 4 compares the versions of CC models with and without the freezer option. The metric values for the two DenseNet 121-based models are similar, with lower bounds for accuracy and balanced accuracy greater than 0.85. For models based on AlexNet, the values of the metrics are slightly lower, which is only statistically significant for precision 1 and recall 0. The corresponding ROC curves are shown in Figure 5.

### 3.3. Performances of the CQ Models

**Figure 6:**
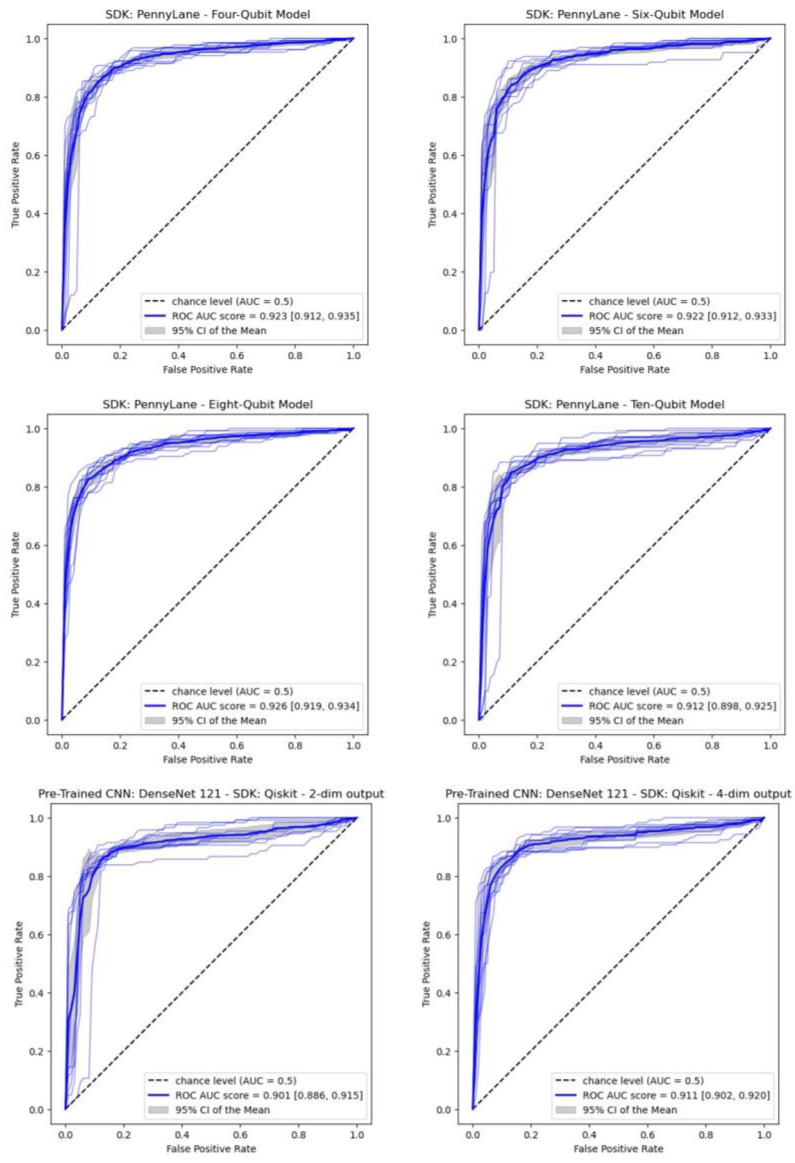
ROC curves obtained by 10-fold cross-validation in five CQ models (test set).

Table 5 lists the metric values for CQ models, all with the freezer option. Compared with the F-Dnet-C model, the AUC score is lower for F-Dnet-P-10q, F-Dnet-Q-4q-2D and F-Dnet-Q-4q-4D (*p*-value < 0.01). The corresponding ROC curves are shown in Figure 6.

### 3.4. Grad-CAM++ Analysis

**Figure 7:**
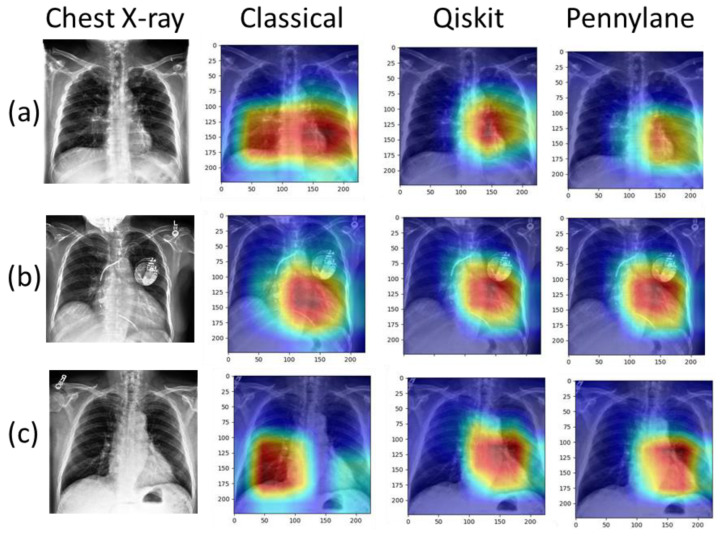
Original CXRs (left) along with the corresponding Grad-CAM++ heatmaps obtained with the last convolutional layer from the three models compared for trusworthiness. (**a**): Normal heart. Large hot zone including the heart with the CC model, hot zones covering the heart with the CQ models. (**b**): Cardiomegaly and artificial pacemaker. Hot zones covering the heart with the three models. (**c**): Cardiomegaly. Hot zone in the right lung base for the CC model (example of non-trustworthy heatmap), hot zones covering the heart for the CQ models.

The blind examination for classification into trustworthy and non-trustworthy concerns 2190 (3 × 730) heatmaps obtained with the three models: F-Dnet-C, F-Dnet-Q-4q-2D and F-Dnet-P-4q. The results of this classification are presented in Table 6. Overall, trustworthy heatmaps were found in 61% of cases with the CC model, in 94% of cases with the Qiskit-based CQ model (chi-square: *p* < 0.001 vs. CC) and in 92% of cases with the PennyLane-based CQ model (*p* < 0.001 vs. CC). Examples of comparisons between models are given in Figure 7 with one case of a non-trustworthy heatmap.

### 3.5. Normalized Global Effective Dimension

**Figure 8:**
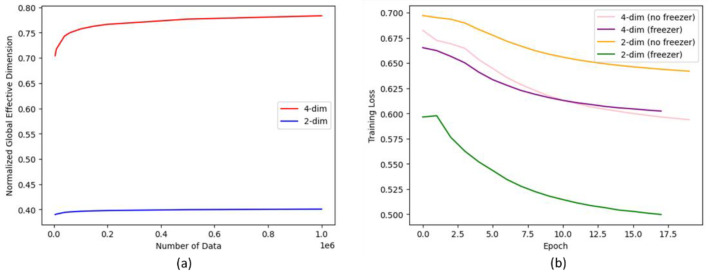
(**a**) NGED for the quantum layer in the classifier in Qiskit four-qubit models with four-dimensional (4-dim) and two-dimensional output (2-dim), each with 24 trainable parameters. (**b**) Training loss curves observed in these models with and without freezer.

Figure 8a shows the NGED curves calculated in a function of the amount of data in the Qiskit-based QC models. We can see that the four-dim output leads to a much higher effective dimension over a wide range. In Figure 8b, the training loss curves observed in these models with and without a freezer are shown. Without the freezer, the two-dim model has higher loss values than those of the four-dim model. With the freezer, the loss values are decreased much more for the two-dim model than for the four-dim model.

## 4. Discussion and Conclusions

In this manuscript, we aimed to study the effect of applying Quantum ML techniques to the detection of cardiomegaly. This is a healthcare problem based on image processing and learning techniques. In particular, we evaluated the performance and trainability of two CC models based on DenseNet-121 and AlexNet, and we modified the DenseNet-121 model by replacing a classical dense layer with a quantum layer leading to CQ models. Following transfer learning training, two different schemas were conducted. In one approach, we froze the weights from the initial layers of the architecture, and in the other approach, we let the weights be updated by our cardiomegaly dedicated data.

We compared the results between the different methods, studied the NGED on the Qiskit-based quantum layer and used Grad-CAM++ to compare the heatmaps obtained from classical and hybrid solutions.

Our protocol required as many common parameters as possible between CC and CQ models to compare them on equal footing. Tuning hyperparameters differently for each model was avoided. We chose twenty epochs after verifying that no sign of an overfitting effect on the test metrics appeared when training CC models up to this number of epochs. Extending this analysis to the CQ models did not seem necessary because the concern about overfitting was triggered essentially by the comparison of training and test ROC curves obtained in the CC models. In addition, this would necessitate considerable time given our computing resources.

The reasons for choosing the F-Dnet-C model as the reference were the generally better test metrics for the versions with the freezer than those without the freezer and the superiority of DenseNet121 over AlexNet for the same criterion via ten-fold cross-validation. Using this approach, the PennyLane-based CQ models up to eight qubits, revealing no statistical differences for any metric compared to the reference. The same was observed for the Qiskit-based models, except that the AUC score was lower. With the exception of the AlexNet-based CC models, the metric values we obtained from the 70/30 train–test split were, in general, very similar to those found via ten-fold cross-validation. Whereas the latter method is one of those recommended for comparing performance in the ML diagnosis of medical images [40], the former retains its interest in exploring a wide range of models with multiple options and for specific types of analysis, involving loss curves or salience maps, for example.

Different factors can influence the efficiency of quantum transfer learning. Among them, the quality and compatibility of the pre-trained models can be cited. This factor was only studied here for choosing the classical reference model. The less-performing AlexNet option was not tested for CQ models. We acknowledge that this could be an interesting part of further studies. Another factor is increased complexity. In models with an increasing number of qubits, the metric values remained the same, with the exception of a significantly lower AUC score for ten qubits. We did not test quantum hardware, an important and rapidly evolving factor. Using Qiskit, the dimension of the quantum classifier output was a factor that seemed to influence training. In this case, further experimentation, preferably on hardware, can be contemplated for analyzing the impact on quantum parallelism.

The details of the quantum part of the algorithm deserve some comments. Adding a PQC between two layers of the trainable classifier is a common technique, although not always necessary or effective in all cases of quantum transfer learning. We made this choice because it has proven its usefulness in other similar quantum applications. Using Qiskit, the PyTorch connection allowed a classification task on a simulator and hardware [22], and using Pennylane, the ‘default.qubit’ simulator was used for the implementation of the model in an application involving CT scan images [26]. For adapting activation functions to quantum circuits, one can use quantum gates, such as the SWAP test or the parametrized quantum circuit (PQC), to transform the quantum state before and after applying the activation function. In another option, the output of the quantum circuit is measured, and the measurement result is used as input to a classical activation function. We chose the approach that is integrated into the quantum SDKs that were available to us and that had demonstrated its effectiveness in other applications. For a proper gradient computation when using TorchConnector in Qiskit, the input_gradients parameter must be explicitly set to True. Equations for the resulting default gradients are given in the Opflow section of the Qiskit documentation. For PennyLane, the automatic “best differentiation method” that we used is a prominent built-in feature that is fully described in its documentation. Other options for the gradients could be considered and explored in further works, especially if convergence issues are detected. The simulator parameters of the SDKs can be tested in an initial phase. For example, 10, 100 and 1024 shots improve metric values to the same extent, well beyond 1 shot in Qiskit-based models. A value of 10 is preferred for these models, which require a long training time in the simulator. For PennyLane-based models, the default number of shots is 1024. We acknowledge that the difference in AUC scores between the models built on these different SDKs is most likely due to these shot number discrepancies.

For heatmap analysis, our blinded human classification into trustworthy and non-trustworthy cases was based on well-defined criteria described in the Materials and Methods section. These criteria had to be adapted for the detection of a single large organ inside the thoracic cage, whereas other published ML studies on CXRs address multiple classes of possibly concomitant abnormal findings [12,21,41,42].

The differences observed in the Grad-CAM++ heatmaps obtained from the three models we examined can have several explanations. First, we used the test set CXRs in the review process, which is more representative of what would be obtained in real applications. The trade-off is that non-trustworthy heatmaps are more likely to show up in test cases than in training cases. Second, the classical algorithm is inherently different from those based on quantum SDKs. Moreover, the Qiskit and Pennylane algorithms are not entirely similar, being based on different code and program functions, which explains the difference in heatmaps in the two CQ models. Third, more aberrantly localized salient zones, or multiple ones, are noted in control cases. Indeed, the target, the heart, is by definition larger in cardiomegaly and therefore less often missed. Determining why precisely the quantum approach performs better, in general, would require further investigations. In the face of large amounts of data and parameters [43], satisfactory visualization can be an emergent property of a CQ model confronted with medical images. A high rate of trustworthy heatmaps is a strong argument for the acceptance of quantum ML in diagnostic applications.

We also examined the possible gain in the trainability of the quantum approach. A supervised classifier maps a higher dimensional input feature space to a lower dimensional output class space. This mapping is largely determined by how well the model recognizes the relations between the most relevant latent features and the features that do not contribute to the decision classes [32]. A model learns feature mapping based on the training data, and therefore, measuring the performance of a model on unseen data is extremely important in assessing the risk involved in the model prediction. The measure of how well the model performs on unseen data is termed as the expressibility or the model’s ability to generalize [44].

Expressive power or the generalization bound is a measure that plays an important role in identifying an overfitting or underfitting model and the risk involved in the model prediction. The generalization bound provides the bounding value (threshold) for the risk assessment. For efficient assessment, the bounding value should consider the training sample size and the richness of the class predictors output by the model [45]. As QNN combines the principles of classical neural networks and PQC, the generalization bounds applied to the classical networks can be adapted to QNN as well [46].

The NGED is a generalization bound that uses Fisher information. Fisher information describes the geometry of the model parameter space and, in turn, the data distribution [47,48,49]. Fisher information also captures the model’s output sensitivity to changes in the input parameter space, thus providing the relationship mapping between the input data and the model output [18].

In QNN models, the cost function or the network gradient is evaluated in the quantum simulator or hardware; then, a classical optimization algorithm trains the parameters of the PQC to minimize the loss or cost of the function [50]. A flat loss landscape or the barren plateau effect, therefore, largely affects the parameter optimization and, hence, the trainability of the QNN models. A barren plateau could be induced either by the noisy quantum hardware or could be circuit-induced (due to random parameter initialization) [51].

In our experiments, we found that the best training performance was obtained in the case of the two-dimensional CQ model, even if the NGED was smaller when compared with the four-dimensional mode. This counterintuitive behavior could be explained based on the fact that we only measured the effective dimension from the quantum layer, or it could be based on the model complexity from the four-dimensional architecture. Nonetheless, we are planning to study the NGED influence on model performance and trainability. In particular, this would help to evaluate the barren plateau effect. Graphical visualization of the algorithm convergence and a comparison of loss curves obtained from simulator vs. real quantum devices would complete such experimental work. This would help set up and evaluate strategies to mitigate the barren plateau effect, e.g., by modifying the quantum ansatz or acting on the training parameters.

As a limitation, the high number of trainable parameters in the classical classifier currently does not allow us to compare the CC and CQ approaches in terms of trainability based on the global effective dimension. Indeed, this would require a very powerful computational resource.

Our findings help define future directions for algorithm improvement, hardware testing, and implementation in a real-life medical environment.

We believe the models can be improved by applying fine-tuning before/after the first two steps with the freezer for a better accuracy outcome [52], tuning the hyper-parameters and having a better-fitted quantum circuit in the quantum layer [22]. Another possibility is applying quantum convolutional and max pool [53]. One can also consider testing the hybrid approach on smaller pre-trained models, such as Resnet34 or Swin Transformer [26,54].

A further step would be to test the CQ models on noisy simulators and NISQ devices. The first approach is easily accessible but remains a delicate task [55] that does not necessarily reflect the performance when using the hardware, which improves itself rapidly. Although even more challenging [56], the evaluation of these models on real quantum computers in terms of performance, resilience and credible visualization would allow us to better affirm their feasibility in real situations. In this process, another full bench would be advised to further study the usability of the models.

Additional research exploring the impact of these CC and QC models in a medical setting may be contemplated. The more nuanced probability of cardiomegaly provided by these types of transfer learning algorithms could have a different clinical meaning from that of the simple reference label or the binary output. This probability is a feature that should be tested against established diagnoses and that should be possibly added to tabular data used by statistical and ML applications in heart failure [4]. We can also use proposed publicly available datasets and open-source software [57] to compare heatmaps generated by different saliency methods in our models to confirm that some hybrid options exhibit better localization performance.

## Figures and Tables

**Table 1:** Final dataset description.

	All	Control	Cardiomegaly
Number of Patients:	2436	1225	1211
Age:	58.6 ± 17.7	54.4 ± 17.2	62.9 ± 17.1 ***
Gender Male:	1520 (62%)	794 (65%)	726 (59%) *
One or Several Other Findings:	1474 (61%)	559 (46%)	915 (75%) ***
Enlarged Cardiomediastinum	293 (12%)	151 (12%)	142 (12%)
Lung Opacity	727 (30%)	266 (22%)	461 (38%) ***
Lung Lesion	191 (8%)	91 (7%)	100 (8%)
Edema	633 (26%)	133 (11%)	500 (41%) ***
Consolidation	647 (27%)	315 (26%)	332 (27%)
Pneumonia	244 (10%)	83 (7%)	161 (13%) ***
Atelectasis	405 (17%)	139 (11%)	266 (22%) ***
Pneumothorax	377 (15%)	215 (18%)	162 (13%) **
Pleural Effusion	1054 (43%)	435 (36%)	619 (51%) ***
Pleural Other	135 (6%)	46 (4%)	89 (7%) ***
Fracture	119 (5%)	59 (5%)	60 (5%)
Support Devices	558 (23%)	226 (18%)	332 (27%) ***

Significant differences between cardiomegaly and control by independent two-sample *t*-test for age and chi-squared contingency for the other features are indicated in the right column. * *p* < 0.05, ** *p* < 0.01, ***: *p* < 0.001.

**Table 2:** Metrics.

	Abbreviation in Tables	Synonyms
AUC score	AUC	---
Accuracy	Acc	---
Balanced Accuracy	B Acc	---
Precision 0	Prec 0	Negative predictive value
Precision 1	Prec 1	Positive predictive value
Recall 0	Rec 0	Specificity
Recall 1	Rec 1	Sensitivity

**Table 3:** Summary of model features.

Model Name	Training with Freezer	Pre-Trained CNN	SDK	*n*	*D*
F-Dnet-C	YES	DenseNet-121	None	---	---
F-Axnet-C	YES	AlexNet	None	---	---
F-Dnet-P-4q	YES	DenseNet-121	PennyLane	4	---
F-Dnet-Q-4q-2D	YES	DenseNet-121	Qiskit	4	2
F-Dnet-Q-4q-4D	YES	DenseNet-121	Qiskit	4	4
N-Dnet-C	NO	DenseNet-121	None	4	--
N-Axnet-C	NO	AlexNet	None	---	--
N-Dnet-P-4q	NO	DenseNet-121	PennyLane	---	--
N-Dnet-Q-4q-2D	NO	DenseNet-121	Qiskit	4	2
N-Dnet-Q-4q-4D	NO	DenseNet-121	Qiskit	4	4
F-Dnet-P-6q	YES	DenseNet-121	PennyLane	6	--
F-Dnet-P-8q	YES	DenseNet-121	PennyLane	8	--
F-Dnet-P-10q	YES	DenseNet-121	PennyLane	10	--

*n*: number of qubits, *D*: output dimension of the classifier.

**Table 4:** Metrics by 10-fold cross-validation in CC models.

Model	AUC	Acc	B Acc	Prec 0	Prec 1	Rec 0	Rec 1
F-Dnet-C	0.931 [0.923, 0.939]	0.863 [0.853, 0.873]	0.862 [0.852, 0.873]	0.845 [0.831, 0.858]	0.884 [0.870, 0.897]	0.892 [0.883, 0.902]	0.832 [0.812, 0.853]
F-Axnet-C	0.933 [0.925, 0.940]	0.858 [0.852, 0.863]	0.858 [0.853, 0.864]	0.858 [0.843, 0.873]	0.858 * [0.835, 0.880]	0.860 * [0.838, 0.881]	0.857 [0.844, 0.870]
N-Dnet-C	0.934 [0.926, 0.942]	0.863 [0.855, 0.870]	0.862 [0.855, 0.870]	0.841 [0.828, 0.853]	0.889 [0.871, 0.908]	0.897 [0.876, 0.917]	0.828 [0.813, 0.843]
N-Axnet-C	0.921 [0.909, 0.933]	0.849 [0.836, 0.863]	0.850 [0.835, 0.864]	0.850 [0.834, 0.866]	0.848 * [0.825, 0.872]	0.851 * [0.830, 0.872]	0.848 [0.830, 0.865]

Differences from F-Dnet-C (paired *t*-test) are indicated by * *p* < 0.05.

**Table 5:** Metrics by 10-fold cross-validation in CQ models.

Model	AUC	Acc	B Acc	Prec 0	Prec 1	Rec 0	Rec 1
F-Dnet-P-4q	0.923 [0.912, 0.935]	0.862 [0.852, 0.872]	0.860 [0.849, 0.871]	0.832 [0.815, 0.848]	0.902 [0.883, 0.920]	0.910 [0.890, 0.931]	0.810 [0.779, 0.841]
F-Dnet-P-6q	0.922 [0.912, 0.933]	0.860 [0.843, 0.878]	0.860 [0.841, 0.878]	0.844 [0.822, 0.865]	0.882 [0.855, 0.909]	0.890 [0.863, 0.918]	0.829 [0.797, 0.862]
F-Dnet-P-8q	0.926 [0.919, 0.934]	0.862 [0.852, 0.873]	0.862 [0.851, 0.873]	0.844 [0.827, 0.861]	0.887 [0.860, 0.913]	0.893 [0.866, 0.920]	0.830 [0.803, 0.858]
F-Dnet-P-10q	0.912 ** [0.898, 0.925]	0.860 [0.849, 0.871]	0.861 [0.850, 0.872]	0.839 [0.816, 0.863]	0.888 [0.860, 0.917]	0.897 [0.869, 0.924]	0.826 [0.798, 0.853]
F-Dnet-Q-4q-2D	0.901 ** [0.886, 0.915]	0.867 [0.859, 0.875]	0.866 [0.858, 0.874]	0.845 [0.830, 0.860]	0.896 [0.877, 0.914]	0.901 [0.874, 0.928]	0.831 [0.807, 0.855]
F-Dnet-Q-4q-4D	0.911 ** [0.902, 0.920]	0.867 [0.859, 0.875]	0.867 [0.859, 0.876]	0.845 [0.829, 0.861]	0.894 [0.879, 0.909]	0.902 [0.887, 0.917]	0.832 [0.814, 0.850]

Differences from F-Dnet-C (paired *t*-test) are indicated by ** *p* < 0.01.

**Table 6:** Grad-CAM++ image analysis.

Heatmap Pattern	Label	CC Model	CQ Model (Qiskit)	CQ Model (PennyLane)
Trustworthy	Control	209	354	342
	Cardiomegaly	237	330	326
	Total	446 (61%)	684 (94%)	668 (92%)
Non-trustworthy	Control	160	15	27
	Cardiomegaly	124	31	35
	Total	284 (39%)	46 (6%)	62 (8%)

**Figure A1:**
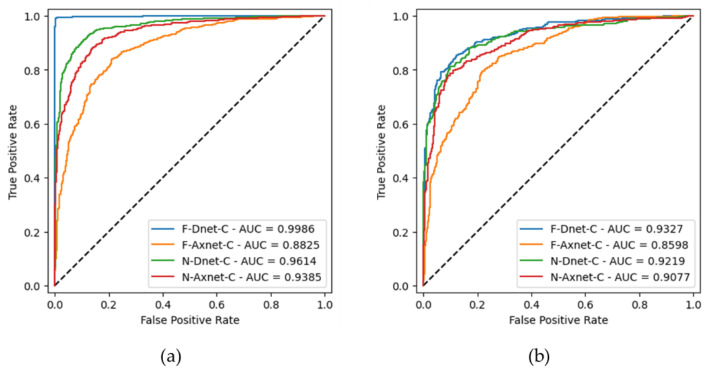
ROC curves for the CC models by 70/30 train–test split: (**a**) Training set. (**b**) Test set.

**Figure A2:**
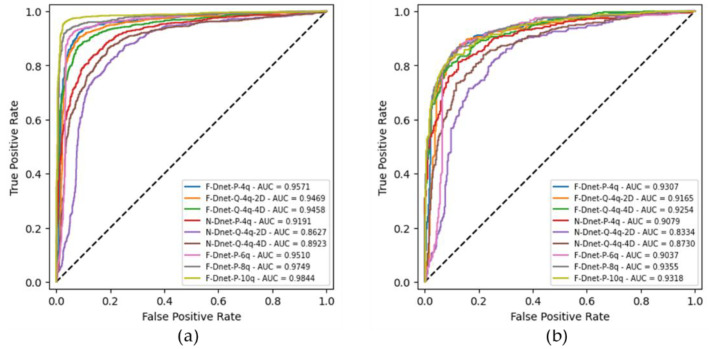
ROC curves for the QC models by 70/30 train–test split: (**a**) Training set. (**b**) Test set.

**Figure A3:**
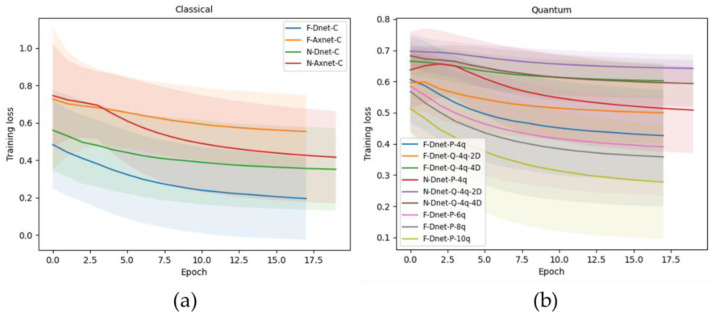
Training loss curves and standard deviation for the CC (**a**) and QC (**b**) models.

**Figure A4:**
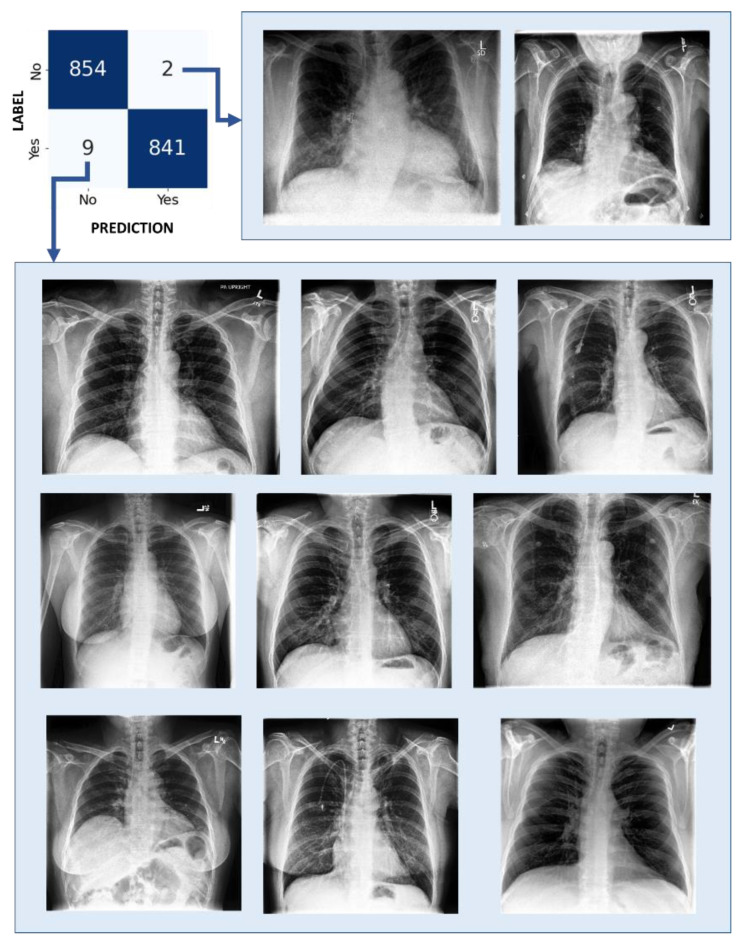
Upper left: confusion matrix for the training set observed for the CC model with Densenet 121 as a feature extractor. Upper right box: two CXRs labeled as control and predicted cardiomegaly. Lower box: 9 CXRs labeled as cardiomegaly and predicted control.

## Data Availability

CVS files with paths to the images and the code used in the experiments are available online: https://github.com/quantum-ai-for-cardiac-imaging/cardiomegaly-chest-x-ray (accessed on 31 March 2023). The images can be downloaded from https://stanfordaimi.azurewebsites.net/datasets/8cbd9ed4-2eb9-4565-affc-111cf4f7ebe2 (accessed on 21 June 2023) after login and agreeing to the Stanford University Dataset Research Use Agreement.

## Appendix A

Table A1 lists the metric values observed in the thirteen experiments. Figure A1 and Figure A2 display the ROC curves observed in these models for the training and test sets.

**Table A1:** Metrics for control (0) and cardiomegaly (1) by 70/30 train–test split.

Model Name	AUC	Acc	B Acc	Prec 0	Prec 1	Rec 0	Rec 1
F-Dnet-C	0.933	0.864	0.865	0.861	0.868	0.873	0.856
F-Axnet-C	0.860	0.785	0.785	0.785	0.785	0.791	0.778
F-Dnet-P-4q	0.931	0.862	0.862	0.885	0.840	0.835	0.889
F-Dnet-Q-4q-2D	0.917	0.862	0.862	0.838	0.889	0.900	0.823
F-Dnet-Q-4q-4D	0.925	0.842	0.842	0.790	0.921	0.938	0.745
N-Dnet-C	0.922	0.853	0.853	0.823	0.892	0.905	0.801
N-Axnet-C	0.908	0.822	0.822	0.839	0.806	0.802	0.842
N-Dnet-P-4q	0.908	0.832	0.831	0.787	0.894	0.913	0.748
N-Dnet-Q-4q-2D	0.833	0.779	0.780	0.810	0.754	0.737	0.823
N-Dnet-Q-4q-4D	0.873	0.804	0.804	0.788	0.822	0.837	0.770
F-Dnet-P-6q	0.904	0.866	0.865	0.841	0.895	0.905	0.825
F-Dnet-P-8q	0.936	0.858	0.857	0.818	0.911	0.924	0.789
F-Dnet-P-10q	0.932	0.860	0.860	0.850	0.871	0.878	0.842

Table A2 contains approximate calculation times per epoch for all models that we trained. For the models with Qiskit-based QPC, we used a 13,700 k CPU, and we used a Cuda GPU for the other models.

**Table A2:** Processing units and calculation times per epoch.

Model Name	Processing Unit	Calculation Time per Epoch
F-Dnet-C/N-Dnet-C	GPU	1 min
F-Axnet-C/C-Axnet-C	GPU	5 s
F-Dnet-P-4q/N-Dnet-P-4q	GPU	3 min
F-Dnet-Q-4q-2D/N-Dnet-Q-4q-2D	GPU and CPU	11 min
F-Dnet-Q-4q-4D/N-Dnet-Q-4q-4D	GPU and CPU	11 min
F-Dnet-P-6q	GPU	4 min
F-Dnet-P-8q	GPU	5 min
F-Dnet-P-10q	GPU	7 min

When comparing the freezer version to the standard version (ten first experiments listed in Table A1), we observed higher metric values with the freezer option in all cases, except when the feature extractor was the AlexNet CNN. Three additional models based on PennyLane, with 6, 8 and 10 qubits, were tested in the freezer version only.

To better shed light on the superiority of the freezer-enhanced protocol over the standard protocol, we compared the loss curves. In the CC models based on AlexNet (Figure A3a), the shortest pre-trained feature extractor, the freezer slowed down the process, whereas the opposite was true in models based on DenseNet-121. In two of the three four-qubit CQ models (six first curves of Figure A3b), all based on pre-trained DenseNet-121, the process started lower and was accelerated with the freezer in two cases: the Qiskit version with two-dimensional output and the PennyLane version. No notable freezer effect was observed for the Qiskit version with four-dimensional output.

For training (Figure A1a), a very high AUC score was obtained in the F-Dnet-C model. Therefore, we looked at the 11 CXRs with a discrepancy between the label and prediction. (Figure A4). In all these cases, the diagnosis obtained by automated label extraction from free-text reports can certainly be disputed.

To complete our investigations about possible overfitting, ten-fold cross-validation was performed in the CC models with different numbers of epochs (Table A3). In an analysis of variance with the fold, number of epochs and feature extractor as the dependent variables, no significant difference was observed among 10, 15 and 20 epochs for any metric value.

**Table A3:** Metrics by ten-fold cross-validation in CC models for different numbers of epochs. Mean and 95% CI for the mean.

Model *	AUC	Acc	B Acc	Prec 0	Prec 1	Rec 0	Rec 1
F-Dnet-C 10 Epochs	0.929 [0.919, 0.938]	0.862 [0.847, 0.877]	0.861 [0.847, 0.876]	0.843 [0.827, 0.860]	0.884 [0.861, 0.908]	0.892 [0.870, 0.915]	0.831 [0.809, 0.852]
F-Dnet-C 15 Epochs	0.931 [0.921, 0.940]	0.862 [0.850, 0.875]	0.863 [0.850, 0.875]	0.844 [0.820, 0.868]	0.884 [0.861, 0.906]	0.891 [0.870, 0.913]	0.834 [0.810, 0.859]
F-Dnet-C 20 Epochs	0.931 [0.923, 0.939]	0.863 [0.853, 0.873]	0.862 [0.852, 0.873]	0.845 [0.831, 0.858]	0.884 [0.870, 0.897]	0.892 [0.883, 0.902]	0.832 [0.812, 0.853]
F-Axnet-C 10 Epochs	0.928 [0.918, 0.938]	0.851 [0.839, 0.862]	0.851 [0.839, 0.862]	0.848 [0.829, 0.868]	0.854 [0.833, 0.876]	0.856 [0.830, 0.883]	0.845 [0.825, 0.866]
F-Axnet-C 15 Epochs	0.927 [0.911, 0.942]	0.857 [0.840, 0.874]	0.857 [0.839, 0.874]	0.855 [0.834, 0.877]	0.857 [0.839, 0.876	0.860 [0.841, 0.879]	0.853 [0.834, 0.873]
F-Axnet-C 20 Epochs	0.933 [0.925, 0.940]	0.858 [0.852, 0.863]	0.858 [0.853, 0.864]	0.858 [0.843, 0.873]	0.858 [0.835, 0.880]	0.860 [0.838, 0.881]	0.857 [0.844, 0.870]

* The number of epochs includes the 2 epochs for the freezer phase.

## References

[1] Cause-Specific Mortality, 2000–2019. https://www.who.int/data/gho/data/themes/mortality-and-global-health-estimates/ghe-leading-causes-of-death.

[2] Centers for Disease Control and Prevention Heart Disease Facts. https://www.cdc.gov/heartdisease/facts.htm.

[3] Timmis A., Vardas P., Townsend N., Torbica A., Katus H., De Smedt D., Gale C.P., Maggioni A.P., Petersen S.E., Huculeci R. (2022). European Society of Cardiology: Cardiovascular Disease Statistics 2021: Executive Summary. Eur. Heart J. Qual. Care Clin. Outcomes.

[4] Averbuch T., Sullivan K., Sauer A., Mamas M.A., Voors A.A., Gale C.P., Metra M., Ravindra N., Van Spall H.G.C. (2022). Applications of Artificial Intelligence and Machine Learning in Heart Failure. Eur. Heart J. Digit. Health.

[5] Wang W., Wang C.-Y., Wang S.-I., Wei J.C.-C. (2022). Long-Term Cardiovascular Outcomes in COVID-19 Survivors among Non-Vaccinated Population: A Retrospective Cohort Study from the TriNetX US Collaborative Networks. eClinicalMedicine.

[6] Mitra M., Samanta R.K. (2013). Cardiac Arrhythmia Classification Using Neural Networks with Selected Features. Procedia Technol..

[7] Yıldırım Ö., Pławiak P., Tan R.-S., Acharya U.R. (2018). Arrhythmia Detection Using Deep Convolutional Neural Network with Long Duration ECG Signals. Comput. Biol. Med..

[8] Amin H., Siddiqui W.J. (2022). Cardiomegaly. StatPearls [Internet].

[9] Felker G.M., Thompson R.E., Hare J.M., Hruban R.H., Clemetson D.E., Howard D.L., Baughman K.L., Kasper E.K. (2000). Underlying Causes and Long-Term Survival in Patients with Initially Unexplained Cardiomyopathy. N. Engl. J. Med..

[10] Heusch G., Libby P., Gersh B., Yellon D., Böhm M., Lopaschuk G., Opie L. (2014). Cardiovascular Remodelling in Coronary Artery Disease and Heart Failure. Lancet.

[11] Bui A.L., Horwich T.B., Fonarow G.C. (2010). Epidemiology and Risk Profile of Heart Failure. Nat. Rev. Cardiol..

[12] Wang X., Peng Y., Lu L., Lu Z., Bagheri M., Summers R.M. ChestX-ray8: Hospital-Scale Chest X-ray Database and Benchmarks on Weakly-Supervised Classification and Localization of Common Thorax Diseases. Proceedings of the IEEE Conference on Computer Vision and Pattern Recognition (CVPR).

[13] Irvin J., Rajpurkar P., Ko M., Yu Y., Ciurea-Ilcus S., Chute C., Marklund H., Haghgoo B., Ball R., Shpanskaya K. (2019). CheXpert: A Large Chest Radiograph Dataset with Uncertainty Labels and Expert Comparison. arXiv.

[14] Johnson A.E.W., Pollard T.J., Berkowitz S.J., Greenbaum N.R., Lungren M.P., Deng C., Mark R.G., Horng S. (2019). MIMIC-CXR, a De-Identified Publicly Available Database of Chest Radiographs with Free-Text Reports. Sci. Data.

[15] Bressem K.K., Adams L.C., Erxleben C., Hamm B., Niehues S.M., Vahldiek J.L. (2020). Comparing Different Deep Learning Architectures for Classification of Chest Radiographs. Sci. Rep..

[16] Susan S., Kumar A. (2020). The Balancing Trick: Optimized Sampling of Imbalanced Datasets—A Brief Survey of the Recent State of the Art. Eng. Rep..

[17] Joseph C. Brain Facts: A Primer on the Brain and Nervous System. Ed.gov. https://eric.ed.gov/?id=ED340602.

[18] Goodfellow I., Bengio Y., Courville A. Deep Learning. Deeplearningbook.org. https://www.deeplearningbook.org/.

[19] Valverde J.M., Imani V., Abdollahzadeh A., De Feo R., Prakash M., Ciszek R., Tohka J. (2021). Transfer Learning in Magnetic Resonance Brain Imaging: A Systematic Review. J. Imaging.

[20] Mukhlif A.A., Al-Khateeb B., Mohammed M.A. (2022). An Extensive Review of State-of-The-Art Transfer Learning Techniques Used in Medical Imaging: Open Issues and Challenges. J. Intell. Syst..

[21] Matsumoto T., Kodera S., Shinohara H., Ieki H., Yamaguchi T., Higashikuni Y., Kiyosue A., Ito K., Ando J., Takimoto E. (2020). Diagnosing Heart Failure from Chest X-ray Images Using Deep Learning. Int. Heart J..

[22] Abbas A., Sutter D., Zoufal C., Lucchi A., Figalli A., Woerner S. (2021). The Power of Quantum Neural Networks. Nat. Comput. Sci..

[23] Maheshwari D., Garcia-Zapirain B., Sierra-Sosa D. (2022). Quantum Machine Learning Applications in the Biomedical Domain: A Systematic Review. IEEE Access.

[24] Houssein E.H., Abohashima Z., Elhoseny M., Mohamed W.M. (2022). Hybrid Quantum-Classical Convolutional Neural Network Model for COVID-19 Prediction Using Chest X-ray Images. J. Comput. Des. Eng..

[25] Mari A., Bromley T.R., Izaac J., Schuld M., Killoran N. (2020). Transfer Learning in Hybrid Classical-Quantum Neural Networks. Quantum.

[26] Shahwar T., Zafar J., Almogren A., Zafar H., Rehman A.U., Shafiq M., Hamam H. (2022). Automated Detection of Alzheimer’s via Hybrid Classical Quantum Neural Networks. Electronics.

[27] Ovalle-Magallanes E., Avina-Cervantes J.G., Cruz-Aceves I., Ruiz-Pinales J. (2022). Hybrid Classical–Quantum Convolutional Neural Network for Stenosis Detection in X-ray Coronary Angiography. Expert Syst. Appl..

[28] Grote T., Berens P. (2019). On the Ethics of Algorithmic Decision-Making in Healthcare. J. Med. Ethics.

[29] Winter P., Carusi A. (2022). If You’re Going to Trust the Machine, Then That Trust Has Got to Be Based on Something. Sci. Technol. Stud..

[30] Chattopadhay A., Sarkar A., Howlader P., Balasubramanian V.N. Grad-CAM++: Generalized Gradient-Based Visual Explanations for Deep Convolutional Networks. Proceedings of the 2018 IEEE Winter Conference on Applications of Computer Vision (WACV).

[31] Benedetti M., Lloyd E., Sack S., Fiorentini M. (2019). Parameterized Quantum Circuits as Machine Learning Models. Quantum Sci. Technol..

[32] Ravichandran K., Jain A., Rakhlin A. Using Effective Dimension to Analyze Feature Transformations in Deep Neural Networks. Proceedings of the ICML 2019 Workshop on Identifying and Understanding Deep Learning Phenomena.

[33] CheXpert Chest X-rays. https://aimi.stanford.edu/chexpert-chest-x-rays.

[34] Truszkiewicz K., Poręba R., Gać P. (2021). Radiological Cardiothoracic Ratio in Evidence-Based Medicine. J. Clin. Med..

[35] Krizhevsky A. (2014). One Weird Trick for Parallelizing Convolutional Neural Networks. arXiv.

[36] Huang G., Liu Z., Van Der Maaten L., Weinberger K.Q. Densely Connected Convolutional Networks. Proceedings of the 2017 IEEE Conference on Computer Vision and Pattern Recognition (CVPR).

[37] Kingma D.P., Ba J. (2017). Adam: A Method for Stochastic Optimization. arXiv.

[38] Torch Connector and Hybrid QNNs. https://qiskit.org/documentation/machine-learning/tutorials/05_torch_connector.html.

[39] Pedregosa F., Varoquaux G., Gramfort A., Michel V., Thirion B., Grisel O., Blondel M., Müller A., Nothman J., Louppe G. (2018). Scikit-Learn: Machine Learning in Python. arXiv.

[40] Singh V., Pencina M., Einstein A.J., Liang J.X., Berman D.S., Slomka P. (2021). Impact of Train/Test Sample Regimen on Performance Estimate Stability of Machine Learning in Cardiovascular Imaging. Sci. Rep..

[41] Rajpurkar P., Irvin J., Ball R.L., Zhu K., Yang B., Mehta H., Duan T., Ding D., Bagul A., Langlotz C.P. (2018). Deep Learning for Chest Radiograph Diagnosis: A Retrospective Comparison of the CheXNeXt Algorithm to Practicing Radiologists. PLoS Med..

[42] Arun N., Gaw N., Singh P., Chang K., Aggarwal M., Chen B., Hoebel K., Gupta S., Patel J., Gidwani M. (2021). Assessing the Trustworthiness of Saliency Maps for Localizing Abnormalities in Medical Imaging. Radiol. Artif. Intell..

[43] Mangini S., Tacchino F., Gerace D., Bajoni D., Macchiavello C. (2021). Quantum Computing Models for Artificial Neural Networks. EPL Europhys. Lett..

[44] Effective Dimension of Qiskit Neural Networks. https://qiskit.org/documentation/machine-learning/tutorials/10_effective_dimension.html.

[45] Sammut C., Webb G.I. (2011). Encyclopedia of Machine Learning.

[46] Quantum Neural Networks. https://qiskit.org/documentation/machine-learning/tutorials/01_neural_networks.html.

[47] Berezniuk O., Figalli A., Ghigliazza R., Musaelian K. (2020). A Scale-Dependent Notion of Effective Dimension. arXiv.

[48] Rissanen J.J. (1996). Fisher Information and Stochastic Complexity. IEEE Trans. Inf. Theory.

[49] Cover T.M., Thomas J.A. (1991). Elements of Information Theory.

[50] Arrasmith A., Cerezo M., Czarnik P., Cincio L., Coles P.J. (2021). Effect of Barren Plateaus on Gradient-Free Optimization. Quantum.

[51] Wang S., Fontana E., Cerezo M., Sharma K., Sone A., Cincio L., Coles P.J. (2021). Noise-Induced Barren Plateaus in Variational Quantum Algorithms. Nat. Commun..

[52] Hossain B., Iqbal S.M.H.S., Islam M., Akhtar N., Sarker I.H. (2022). Transfer Learning with Fine-Tuned Deep CNN ResNet50 Model for Classifying COVID-19 from Chest X-ray Images. Inform. Med. Unlocked.

[53] Oh S., Choi J., Kim J. A Tutorial on Quantum Convolutional Neural Networks (QCNN). IEEE Xplore. Proceedings of the 2020 International Conference on Information and Communication Technology Convergence (ICTC).

[54] Jiang J., Lin S. (2022). COVID-19 Detection in Chest X-ray Images Using Swin-Transformer and Transformer in Transformer. arXiv.

[55] Xu X., Benjamin S., Sun J., Yuan X., Zhang P. (2023). A Herculean Task: Classical Simulation of Quantum Computers. arXiv.

[56] Yang Z., Zolanvari M., Jain R. (2023). A Survey of Important Issues in Quantum Computing and Communications. IEEE Commun. Surv. Tutor..

[57] Saporta A., Gui X., Agrawal A., Pareek A., Truong S.Q.H., Nguyen C.D.T., Ngo V.-D., Seekins J., Blankenberg F.G., Ng A.Y. (2022). Benchmarking Saliency Methods for Chest X-ray Interpretation. Nat. Mach. Intell..

[58] Anis M.S., Abraham H., Adu O., Agarwal R., Agliardi G., Aharoni M., Akhalwaya I.Y., Aleksandrowicz G., Alexander T., Amy M. (2021). Qiskit: An Open-Source Framework for Quantum Computing. https://raw.githubusercontent.com/Qiskit/qiskit/master/Qiskit.bib.

[59] Bergholm V., Izaac J., Schuld M., Gogolin C., Ahmed S., Ajith V., Alam M.S., Alonso-Linaje G., AkashNarayanan B., Asadi A. (2022). PennyLane: Automatic Differentiation of Hybrid Quantum-Classical Computations. arXiv.

[60] Paszke A., Gross S., Massa F., Lerer A., Bradbury J., Chanan G., Killeen T., Lin Z., Gimelshein N., Antiga L. (2019). PyTorch: An Imperative Style, High-Performance Deep Learning Library. http://papers.neurips.cc/paper/9015-pytorch-an-imperative-style-high-performance-deep-learning-library.pdf.

